# Dual Targeting of Akt and mTORC1 Impairs Repair of DNA Double-Strand Breaks and Increases Radiation Sensitivity of Human Tumor Cells

**DOI:** 10.1371/journal.pone.0154745

**Published:** 2016-05-03

**Authors:** Marina Holler, Astrid Grottke, Katharina Mueck, Julia Manes, Manfred Jücker, H. Peter Rodemann, Mahmoud Toulany

**Affiliations:** 1 Division of Radiobiology and Molecular Environmental Research, Department of Radiation Oncology, Eberhard Karls University Tuebingen, Roentgenweg 11, 72076, Tuebingen, Germany; 2 Institute of Biochemistry and Signal Transduction, University Medical Center Hamburg-Eppendorf, Martinistrasse 52, 20246, Hamburg, Germany; The University of Hong Kong, HONG KONG

## Abstract

Inhibition of mammalian target of rapamycin-complex 1 (mTORC1) induces activation of Akt. Because Akt activity mediates the repair of ionizing radiation-induced DNA double-strand breaks (DNA-DSBs) and consequently the radioresistance of solid tumors, we investigated whether dual targeting of mTORC1 and Akt impairs DNA-DSB repair and induces radiosensitization. Combining mTORC1 inhibitor rapamycin with ionizing radiation in human non-small cell lung cancer (NSCLC) cells (H661, H460, SK-MES-1, HTB-182, A549) and in the breast cancer cell line MDA-MB-231 resulted in radiosensitization of H661 and H460 cells *(responders)*, whereas only a very slight effect was observed in A549 cells, and no effect was observed in SK-MES-1, HTB-182 or MDA-MB-231 cells (*non-responders*). In *responder* cells, rapamycin treatment did not activate Akt1 phosphorylation, whereas in *non-responders*, rapamycin mediated PI3K-dependent Akt activity. Molecular targeting of Akt by Akt inhibitor MK2206 or knockdown of Akt1 led to a rapamycin-induced radiosensitization of *non-responder* cells. Compared to the single targeting of Akt, the dual targeting of mTORC1 and Akt1 markedly enhanced the frequency of residual DNA-DSBs by inhibiting the non-homologous end joining repair pathway and increased radiation sensitivity. Together, lack of radiosensitization induced by rapamycin was associated with rapamycin-mediated Akt1 activation. Thus, dual targeting of mTORC1 and Akt1 inhibits repair of DNA-DSB leading to radiosensitization of solid tumor cells.

## Introduction

The mammalian target of rapamycin (mTOR) pathway plays a major role in the regulation of cell growth, proliferation and survival [1, 2]. The serine/threonine kinase mTOR exists in two distinct complexes, mTOR complex-1 (mTORC1) and mTOR complex-2 (mTORC2). S6K1 and 4EBP1 are downstream signaling elements of mTORC1 that promote tumor cell growth by stimulating protein synthesis [2, 3]. Signaling pathways that are upstream or downstream of mTOR are commonly deregulated in human cancers. Therefore, targeting mTOR has been proposed to be a promising approach in cancer therapy [3]. In preclinical studies, a cytostatic effect of mTOR inhibitors has been reported in a variety of cancers [4, 5]. Although data from clinical trials indicate that mTOR targeting improves survival in patients with advanced renal cell carcinoma [6, 7], in many other solid tumor types the response rates and clinical benefits are modest [8]. Rapamycin, an allosteric mTORC1 inhibitor, and its analogs inhibit mTORC1 kinase activity. The limited effectiveness of mTORC1 inhibitors may be due to a lack of complete inhibition of mTORC1 [9] or, more importantly, it may be due to rapamycin-mediated activation of the PI3K/Akt pathway [10]. Physiological activation of the PI3K/Akt/mTORC1 pathway is regulated by a negative feedback mechanism, whereby S6K1-mediated phosphorylation leads to inactivation of insulin receptor substrate 1 (IRS1) and consequently to decreased PI3K/Akt activity [11, 12]. The inhibition of mTORC1 by rapamycin abrogates this feedback regulation, leading to PI3K-dependent Akt phosphorylation [13, 14].

Preclinical studies have indicated that the activation of Akt1 is associated with radiotherapy resistance [15–17]. The Akt protein, and, especially, the Akt1 isoform, promotes survival in cells after exposure to ionizing radiation (IR) by accelerating the repair of DNA-DSBs [18–22]. In cells that have been exposed to radiation, Akt1 and DNA-dependent protein kinase catalytic subunit (DNA-PKcs) form a functional complex, which serves to initiate DNA-DSBs repair through non-homologous end joining (NHEJ) [23]. Thereafter, Akt plays a pivotal role in the recruitment of the Akt1/DNA-PKcs complex to DNA duplex ends that have been marked by Ku dimers. Moreover, Akt1 promotes DNA-PKcs kinase activity, which is a necessary step for the progression of DNA-DSBs repair. Akt1-dependent DNA-PKcs kinase activity stimulates autophosphorylation of DNA-PKcs at S2056, which is needed for efficient DNA-DSBs repair and for the release of DNA-PKcs from a damage site. [23].

The goal of this study was to determine the influence of Akt activity on mTORC1 inhibition-induced radiosensitization. Our data indicate for the first time that in tumor cells, which are not radiosensitized by rapamycin, the inhibition of mTORC1 by rapamycin leads to enhanced activation of Akt, especially Akt1 isoform, and consequently accelerated repair of IR-induced DNA-DSBs. Thus, the dual targeting of mTORC1 and Akt represents an efficient method of promoting the radiosensitization that is induced by rapamycin and thereby improves radiation response of solid tumors.

## Materials and Methods

### Antibodies and Inhibitors

Antibodies against phospho-mTOR (S2448, Cat# 2971), mTOR, phospho-S6 (S235/236, Cat# 4856), S6, phospho-Akt1 (S473, Cat# 9271), phospho-Akt1 (T308, Cat# 9275), phospho-GSK-3α/β (S21/9, Cat# 8566), GSK-3α/β (Cat# 5676), phospho-PRAS40 (T246, Cat# 2997), PRAS40 (Cat# 2691), P-Akt2 (S474, Cat# 8599), Akt2 (Cat# 5239) and GAPDH (Cat# 2118) were purchased from Cell Signaling Technology (Frankfurt, Germany). Rapamycin was obtained from Enzo Life Science (Loerrach, Germany). The PI3K inhibitor LY294002 was purchased from Calbiochem (Schwalbach, Germany). The Akt inhibitor MK2206 was purchased from Selleck Chemicals (Munich, Germany). SMART pool siRNA against AKT1 (Cat. No. M-0030000-03) and AKT2 (Cat. No. M-003001-02), as well as a non-targeting siRNA (Cat. No. D-001810-10) were purchased from Thermo Scientific Dharmacon (Bonn, Germany). Anti-Akt1 antibody, anti-phospho-histone H2AX (S139) antibody and the Alexa Fluor 488 conjugated secondary antibody have been previously described [16, 23].

### Cell Lines

The human lung carcinoma cell lines A549, SK-MES-1, HTB-182, H661 and H460 and the breast cancer cell line MDA-MB-231 were used. Cells were cultured in either DMEM (A549, SK-MES-1, HTB-182, MDA-MB-231) or RPMI-1640 (H661 and H460). All cells were routinely supplemented with 10% fetal calf serum and 1% penicillin-streptomycin and incubated in a humidified atmosphere of 93% air/ 7% CO_2_ at 37°C. PLKO.1-puro vectors encoding either AKT1 or scrambled-shRNA were purchased from Sigma-Aldrich (Taufkirchen, Germany). The generation of pseudotyped lentiviruses and the measurement of transduction were performed as described previously [24]. To generate stable AKT1 knockdown cells, MDA-MB-231 cells were transduced with vectors containing either AKT1 or scrambled shRNA, and colonies were selected following the addition of puromycin (Sigma-Aldrich, Taufkirchen, Germany) to culture medium at a final concentration of 1.5 μg/ml for at least one week. SK-MES-1 and HTB-182 were kindly provided by Dr. Nils Cordes, OncoRay—National Center for Radiation Research in Oncology, Faculty of Medicine and University Hospital Carl Gustav Carus, Technische Universitaet Dresden, Germany. H460 and H661 were received from Dr. Ali Sak, Department of Radiotherapy, University Hospital Essen, Essen, Germany. A549 cells stably transfected with NHEJ reporter constructs and pDsRed-I-SceI expression vector were kindly provided by Dr. Malte Kriegs and Dr. Wael Y. Mansour (Laboratory of Radiobiology and Experimental Radiooncology, University Medical Center Hamburg-Eppendorf, Hamburg, Germany), respectively.

### Inhibitor Treatment

Rapamycin, MK2206 and LY294002 were independently dissolved in DMSO to create 10 mM stock solutions, which were stored at -20°C. The inhibitor solutions were diluted in cell media to appropriate working concentrations just prior to use. Control cells received media that contained appropriate concentrations of the solvent DMSO.

### Proliferation Assay

To test the antiproliferative effect of rapamycin, 3 x 10^4^ cells were seeded in 6 cm tissue culture plates and grown overnight before being treated with either DMSO or rapamycin at the indicated concentrations. At days 2, 3 and 4 after treatment, cells were collected by trypsinization and counted; growth curves were graphed. The cell population doubling time (PDT) was calculated in control cells and in cells treated with rapamycin. The antiproliferative effect of rapamycin was calculated by determining the ratio of the PDT in rapamycin treated cells to the PDT in control cells.

### γ-H2AX Foci Assay, Colony Formation Assay, siRNA Transfection and Western Blot Analysis

γ-H2AX foci assay to quantify residual IR-induced DNA-DSBs, clonogenic cell survival following exposure to ionizing radiation, siRNA transfection and Western blot analysis were performed as described previously [16, 21, 23].

To determine the radiosensitizing effects of the applied inhibitors, a dose modification factor (DMF) was calculated by dividing the irradiation dose that led to 37% survival in control cells to the irradiation dose that led to 37% survival in inhibitor-treated cells. A DMF > 1.00 indicates radiosensitization.

### Non-Homologous End-Joining Repair Assay

A549 cells stably transfected with NHEJ reporter constructs were used [25, 26]. Cells were transiently transfected with 1 μg of the pDsRed-I-SceI expression vector by using Lipofectamine. Confluent cells were treated with inhibitors 24 h after transfection. The percentage of GFP-positive cells was determined using a flow cytometer after additional 24 h.

### Statistics and Densitometry

Student’s *t*-test was used to compare data between groups. P < 0.05 was considered statistically significant (*P < 0.05; **P < 0.01; ***P < 0.001). Densitometry quantification analyses of the immunoblots were performed with ImageJ 1.44p software (http://imagej.nih.gov/ij/).

## Results

### Anti-Proliferative and Radiosensitizing Effects of Rapamycin in NSCLC Cells

Previously, it has been shown that rapamycin mediates a cell line-dependent antiproliferative effect that is not restricted to tumor cells from specific cancers [27, 28]. In this study, this effect was further analyzed in five NSCLC cell lines: A549, SK-MES-1, HTB-182, H661 and H460. As shown in S1 Fig, rapamycin partially inhibited the proliferation of all the cell lines that were tested (S1 Fig and S1 Table). The data, which are summarized in S1 Table, indicate that a 100 nM concentration of rapamycin increased the population doubling time (PDT) of H661 cells by approximately 67%, whereas in H460 cells this effect was only approximately 9%. The rapamycin-induced prolongation of the PDT was 40%, 26% and 21% in A549, SK-MES-1 and HTB-182, respectively. A similar antiproliferative effect was observed when the NSCLC cell lines were treated with concentrations of 200 or 500 nM rapamycin.

**Fig 1:**
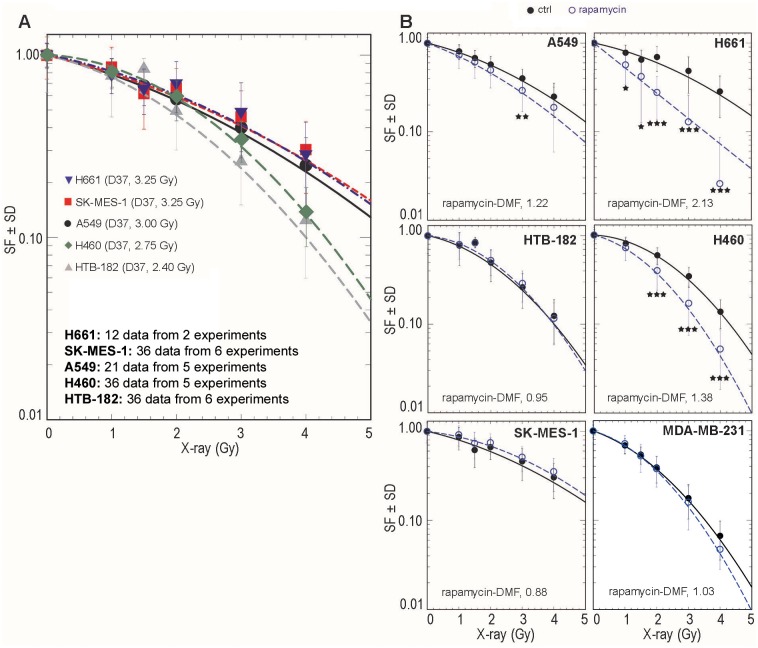
Radiosensitizing effect of rapamycin in NSCLC and breast cancer cells. **(A)** Cells were plated in culture dishes and were irradiated after 24 hours. Each data point shown in Fig 1A represents the mean SF ± SD of indicated number of data obtained from indicated biologically independent experiments. (**B)** Twenty-four hours after pre-plating, cells were treated with rapamycin (100 nM) for 2 h and irradiated with single doses of 0 to 4 Gy. Following irradiation, cultures were incubated at 37°C to facilitate colony growth. After approximately 10–12 days, colonies were stained and counted. Asterisks indicate significant radiosensitizing effects (*, P < 0.05; **, P < 0.01; ***, P < 0.001). DMF: Dose modification factor.

Although rapamycin partially reduced the proliferation of NSCLC cells, there is still a rationale for combining rapamycin with other approaches of cancer therapy, such as radiotherapy. Thus, we further investigated the efficacy of rapamycin in producing radiosensitization by examining NSCLC cells that are known to have differential intrinsic sensitivities to radiation (Fig 1A). As indicated by D_37_ values (dose required to reduce survival to 37%), H661 (D_37_, 3.25 Gy), SK-MES-1 (D_37_, 3.25 Gy) and A549 (D_37_, 3.00 Gy) cells were more radioresistant than H460 (D_37_, 2.75 Gy) and HTB-182 (D_37_, 2.40 Gy) cells. Interestingly, a 100 nM concentration of rapamycin significantly radiosensitized H661 cells, with a dose modification factor (DMF) (ratio of irradiation dose for 37% survival in control cells to irradiation dose for 37% survival in inhibitor-treated conditions) of 2.13, and H460 (DMF, 1.38) cells, whereas only a slight effect was observed in response to rapamycin treatment in A549 (DMF, 1.22) cells. Rapamycin at the concentration of 100 nM, which is significantly higher than the dose needed to inhibit S6K phosphorylation via mTOR, as described in human soft tissue sarcoma cell lines [29], did not radiosensitize HTB-182 (DMF, 0.95), SK-MES-1 (DMF, 0.88) or MDA-MB-231 cells (DMF, 1.03) (Fig 1B). Based on these results, H661 and H460 cells were characterized as *responder* cell lines, and A549, HTB-182, SK-MES-1 and MDA-MB-231 cells as *non-responder* cell lines.

### Rapamycin Treatment Induces Akt1 Activation in a Cell Line-Dependent Manner

**Fig 2:**
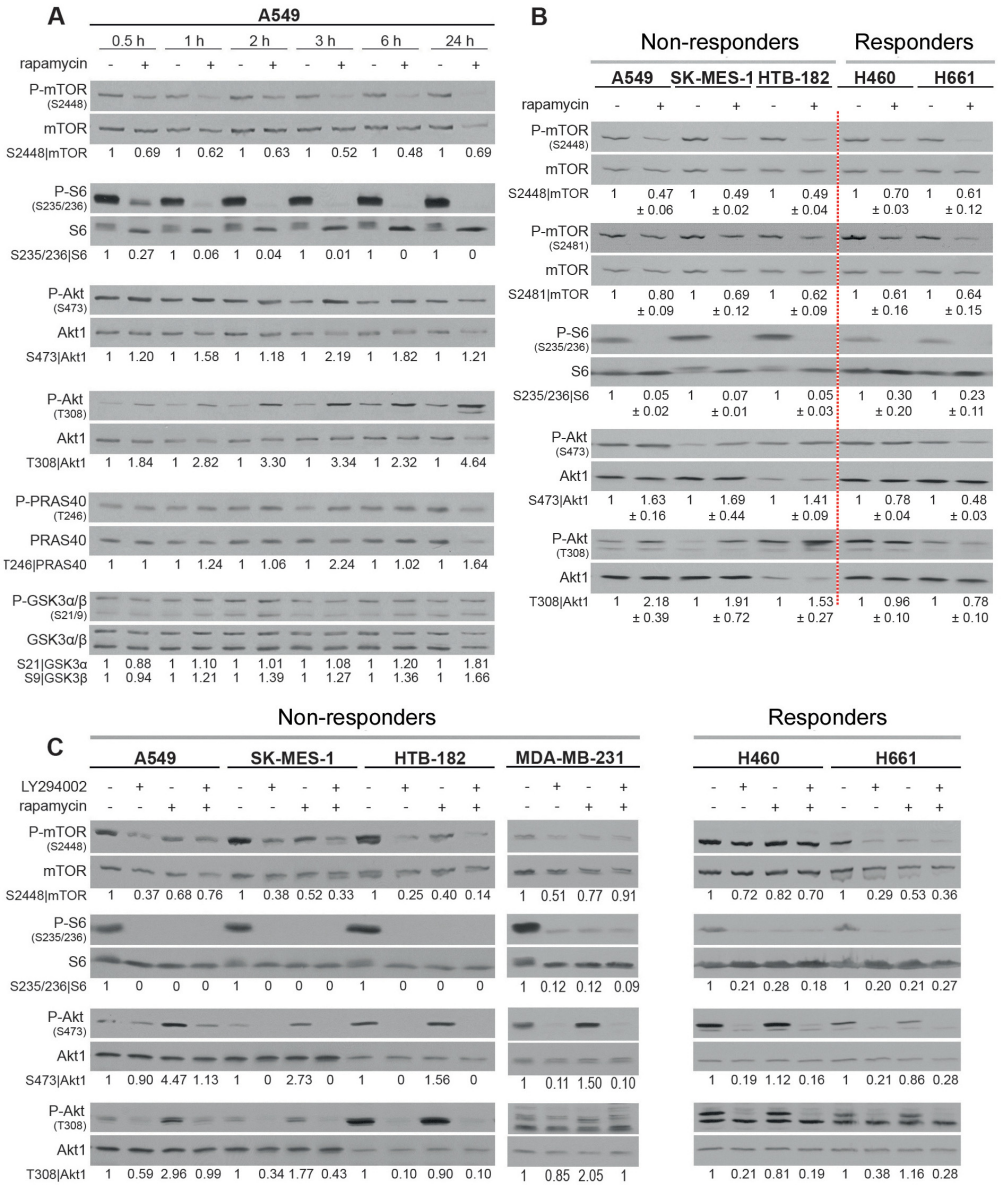
Rapamycin treatment induces Akt1 activation through PI3K in a cell line-dependent manner. Cells were treated with rapamycin (100 nM) for the indicated time points **(A)** or for 6 h **(B)**. (**C)** After 1 h pretreatment with LY294002 (10 μM), cells were treated with rapamycin (100 nM) for 2 hours. Control cells received the appropriate concentrations of DMSO. Thereafter, protein samples were isolated and the phosphorylation patterns of mTOR, S6, Akt, PRAS40 and GSK3α/β were analyzed by Western blotting. Blots were stripped and incubated with antibodies against total proteins. Densitometry values represent the ratio of phosphorylated protein to total protein, which was normalized to 1 for the control condition. Densitometry values represented in part B are at least from 3 independent experiments.

**Fig 3:**
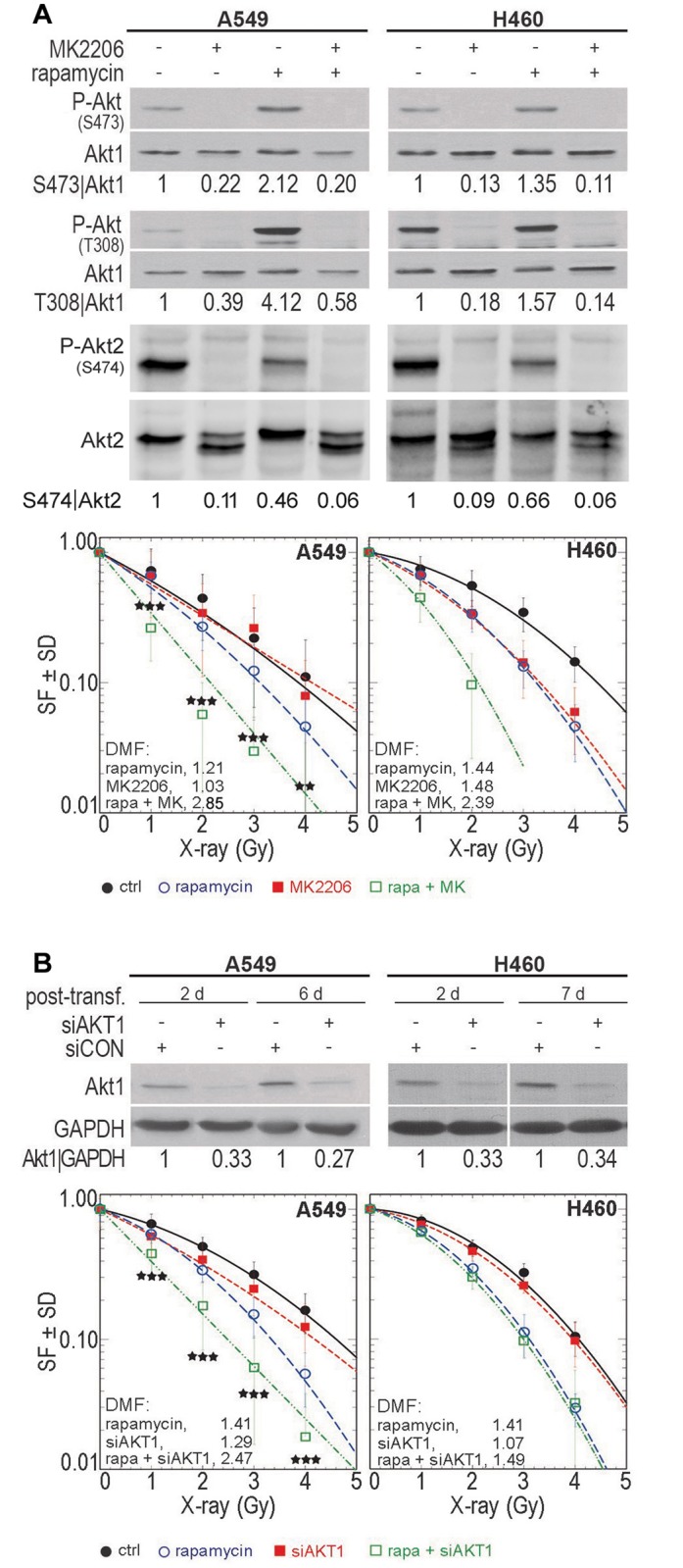
Akt targeting promotes the radiosensitizing effect of rapamycin in *non-responder* cells. **(A)** Cells were treated with MK2206 (A549: 5 μM; H460: 2.5 μM) for 1 h, followed by treatment with rapamycin (100 nM) for 2 h. Control cells received the appropriate concentrations of DMSO. Thereafter, P-Akt (S473, T308) and P-Akt2 (S474) levels were analyzed in protein samples by Western blotting. Blots were stripped and incubated with anti-Akt1 or anti-Akt2 antibodies. The densitometry data represent the mean ratio of phosphorylated Akt1 to Akt1 based on 2 biologically independent experiments; the control conditions were normalized to 1. The densitometry values for phospho-Akt2 represent the mean ratio of phospho-S474 to Akt2 normalized to 1 for the control condition. For colony formation assays, 24 h after pre-plating, cells were treated with MK2206 (A549: 5 μM; H460: 2.5 μM) for 1 h, followed by treatment with rapamycin (100 nM) for 2 h. Control cells received the appropriate concentrations of DMSO. The cultures were irradiated after rapamycin treatment and incubated for colony growth. Data represent the mean SF ± SD of 18 data from three biologically independent experiments in A549 cells and of 12 data from two biologically independent experiments in H460 cells. In *non-responder* A549 cells, asterisks indicate a significant difference between the radiosensitizing effect produced by the combination of MK2206 and rapamycin compared to the effects of single treatment with rapamycin alone (**, P < 0.01; ***, P < 0.001). **(B)** Cells were plated in culture dishes and were transfected with non-target-siRNA (siCON) or AKT1-siRNA (siAKT1) after 24 hours at concentrations of 50 nM (A549) and 150 nM (H460). At the indicated time points after transfection, Akt1 knockdown efficiency was tested by Western blotting. GAPDH was used as a loading control. Densitometry values represent the mean ratio of Akt1 to GAPDH from three independent experiments; the control conditions were normalized to 1. In parallel, 2 days after transfection, cells were trypsinized and seeded for clonogenic assays. Twenty-four hours later, cells were treated with rapamycin (100 nM) for 2 hours, followed by irradiation with single doses of 0 to 4 Gy. The clonogenic assay was performed as described in *Materials and Methods*. Data represent the mean SF ± SD of four biologically independent experiments (21 data) in A549 cells and 6 parallel experiments in H460 cells. In *non-responder* A549 cells, asterisks indicate a significant difference between the radiosensitizing effect produced by the combination of AKT1-siRNA with rapamycin compared to the effects of single treatment with rapamycin alone (***, P < 0.001). DMF: Dose modification factor.

It is known that targeting mTORC1 with rapamycin results in the activation of Akt [14], which is involved in the post-irradiation survival of NSCLC [30]. Thus, we hypothesized that the differential radiosensitizing effect of rapamycin (Fig 1B) might be due to a differential effect on Akt activity. We found the first evidence of an association between rapamycin-induced Akt activity and a lack of radiosensitization in the *non-responder* A549 cell line. As shown in Fig 2A, rapamycin (100 nM) markedly attenuated the transphosphorylation of mTOR (S2448) and its downstream molecule S6 (S235/236) in a time-independent manner. This decrease occurred within 30 minutes of rapamycin treatment and was maintained for up to 24 hours. Interestingly, in parallel to the inhibition of the mTORC1 pathway, treatment with rapamycin resulted in a sustained increase in Akt1 phosphorylation at S473 and T308 (Fig 2A). Furthermore, analysis of the phosphorylation patterns of the Akt substrates GSK3α/β (S21/9) and PRAS40 (T246) indicated that, in addition to phosphorylating Akt1, rapamycin slightly induced Akt1 kinase activity. The concomitant rapamycin-induced Akt1 phosphorylation/activation and absence of radiosensitization was further investigated in the *non-responder* NSCLC cell lines SK-MES-1 and HTB-182. As shown in Fig 2B, rapamycin treatment blocked the phosphorylation of mTOR at its transphosphorylation (S2448) and autophosphorylation sites (S2481), and it also markedly induced Akt1 phosphorylation. Similar to the data shown in Fig 2A, rapamycin stimulated Akt1 phosphorylation in A549 cells. In contrast, in *responder* H460 and H661 cells, the phosphorylation of Akt1 at both S473 and T308 was slightly reduced following rapamycin treatment (Fig 2B). Moreover, Akt1 phosphorylation (S473, T308) that was mediated by rapamycin was markedly blocked by the PI3K inhibitor LY294002 (10 μM) in the *non-responder* A549, SK-MES-1 and HTB-182 cell lines (Fig 3C). In a similar experiment performed in the *responder* H460 and H661 cell lines, LY294002 was shown to block basal phosphorylation of Akt1 at S473 and T308. Additionally, rapamycin-induced PI3K-dependent Akt1 activity and a lack of radiosensitization were concurrently observed in the breast cancer cell line MDA-MB-231 (Figs 1B & 2C).

### Akt Targeting Promotes the Radiosensitizing Effect of Rapamycin in Non-Responder Cells

The specific function of Akt activity in rapamycin-mediated radiosensitization was investigated in *non-responder* cells A549 and *responder* cells H460. Selection of these cell lines is based on the similarities in the mutational status of EGFR, TP53 and K-RAS, which might affect Akt signaling. Both selected cell lines have wild type EGFR, wild type TP53 and mutated K-RAS. In *non-responder* A549 cells, the rapamycin-mediated stimulation of phosphorylation of Akt1 at S473 and T308 was blocked by the Akt inhibitor MK2206 (5 μM). Similarly, MK2206 (2.5 μM) blocked basal Akt1 phosphorylation in *responder* H460 cells (Fig 3A). In contrast to rapamycin-induced Akt1 phosphorylation (S473 and T308) in *non-responder* cells, phosphorylation of Akt2 (S474) in both *non-responder* (A549) and *responder* (H460) cells was markedly inhibited by rapamycin. In both cell lines, a very strong inhibition of Akt2 phosphorylation was achieved by Akt inhibitor MK2206. Furthermore, the effect of the combined treatment of rapamycin and MK2206 on radiation sensitivity was investigated. As expected, similar to the data shown in Fig 1B, rapamycin exerted a slight radiosensitizing effect on A549 cells (DMF, 1.21) and a strong effect on H460 cells (DMF, 1.44) (Fig 3A). The Akt inhibitor MK2206 had a radiosensitizing effect on H460 cells (DMF, 1.48), but not on A549 cells (DMF, 1.03) (Fig 3A, S2 Fig). A combined treatment of MK2206 and rapamycin enhanced the level of rapamycin-induced radiosensitization in A549 cells (DMF, 2.85) and produced an additive effect in H460 cells (DMF, 2.39) (Fig 3A). Potential of Akt inhibition on improved rapamycin-mediated radiosensitization was investigated in *non-responder* SK-MES-1 cells as well. As shown in S3 Fig, similar to the data shown Fig 1B, rapamycin alone did not induce radiosensitization while Akt inhibition led to a marked radiosensitization. However, radiosensitizing effect of Akt inhibitor in rapamycin treated cells was markedly stronger (S3 Fig).

The specific function of Akt1 in cells that did not undergo radiosensitization in response to rapamycin was further investigated using a siRNA approach. To accomplish this, cells were transfected with AKT1-siRNA and then subjected to clonogenic assays in the presence and absence of rapamycin at 48 hours after transfection. The results indicated that A549 cells that were transfected with AKT1-siRNA (50 nM) exhibited significantly induced radiosensitization (DMF, 1.29) (Fig 3B). The combination of AKT1-siRNA and rapamycin led to improved rapamycin-induced radiosensitization in A549 cells (DMF, 2.47). Likewise, rapamycin-induced radiosensitization was significantly enhanced in *non-responder* MDA-MB-231 cells in which Akt1 was stably knocked down by shRNA (Fig A in S4 Fig). In *responder* H460 cells, the efficiency of AKT1-siRNA was not sufficient (data not shown). By increasing the siRNA concentration to 150 nM, a better Akt1 knockdown was achieved, and it remained stable for up to 7 days post-transfection. Under this condition, AKT1-siRNA alone did not affect the intrinsic level of radiation sensitivity in H460 cells (DMF, 1.07) (Fig 3B). In combination with AKT1-siRNA, the rapamycin-induced radiosensitization of H460 cells was unchanged (Fig 3B).

### Rapamycin-Induced Akt Activation Accelerates the Repair of Radiation-Induced DNA-DSBs

**Fig 4:**
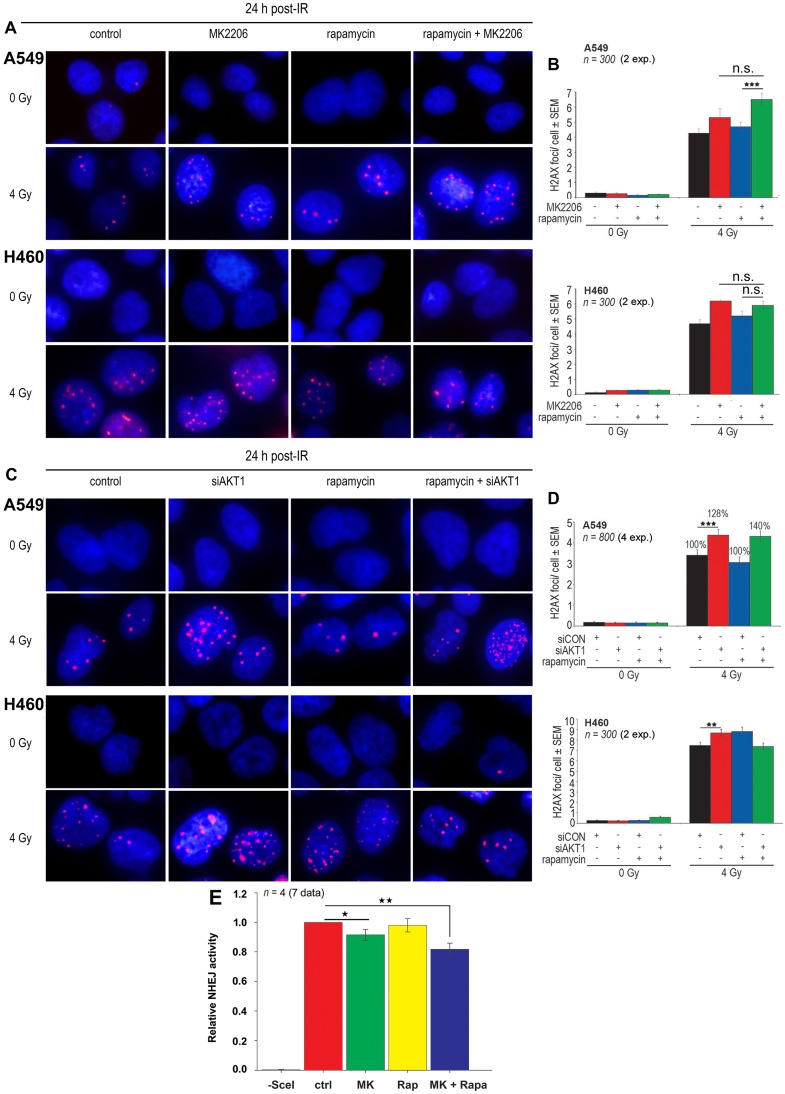
Rapamycin-induced Akt activation accelerates the repair of radiation-induced DNA-DSBs. **(A-B)** Cells grown to confluency on glass slides were treated with MK2206 (A549: 5 μM; H460: 2.5 μM) and rapamycin (100 nM) or were pretreated with MK2206 (A549: 5 μM; H460: 2.5 μM) for 1 hour and followed by treatment with rapamycin (100 nM) for 2 h. **(C-D)** Cells grown on glass slides were transfected with control-siRNA (siCON) or AKT1-siRNA (siAKT1) (A549: 50 nM; H460: 150 nM). Seventy-two hours after transfection, cells were treated with rapamycin (100 nM) for 3 hours. In the experiments described above, control cells received the appropriate concentrations of DMSO. Following the treatment procedures described above, cells were irradiated with the indicated doses of X-ray, and γ-H2AX foci assays were performed 24 h after irradiation, as described in *Materials and Methods*. The frequency of residual γ-H2AX foci was counted in indicated number of cells and experiments (exp.) per treatment condition. The mean number of foci/cell ± SEM was calculated and graphed. Asterisks indicate statistically significant enhancement of residual γ-H2AX foci under the indicated conditions (*, P < 0.05; **, P < 0.01; ***, P < 0.001). (**E**) Data represent the mean value of GFP expression from 7 data obtained from 4 independent experiments after 24 h treatment with DMSO, MK2206 (MK /5 μM), Rapamycin (Rapa / 100 nM) or the combination of MK2206 with rapamycin in the NHEJ assay performed in A549 cells expressing NHEJ assay platform, as described in the *Materials and Methods* section (*, P < 0.05; **, P < 0.01).

Residual DNA double-strand breaks (DNA-DSBs) are crucial for IR-induced cell death. It is known that Akt activity accelerates the repair of DNA-DSBs, leading to radioresistance *in vitro* [31, 32]. Thus, we tested whether rapamycin-mediated Akt activity accelerates the repair of IR-induced DNA-DSBs and leads to enhanced post-irradiation cell survival. Therefore, *non-responder* A549 and *responder* H460 cells were treated with the Akt inhibitor MK2206 in combination with rapamycin and were irradiated with 4 Gy. Compared to the effect produced by MK2206 alone, the combination of rapamycin and MK2206 led to an increased number of residual DNA-DSBs in both the 2 Gy and 4 Gy irradiated *non-responder* A549 cells, but not in *responder* H460 cells as shown by the representative images in Fig 4A and the average number of foci per cell under different treatment conditions in Fig 4B.

In line with previous reports from our laboratory [21, 23], Akt1 knockdown impaired the repair of radiation-induced DNA-DSBs in *non-responder* A549 cells and in *responder* H460 cells, as shown by the significant increase in the number of residual γ-H2AX foci at 24 hours post-irradiation (Fig 4C and 4D). However, after irradiation with 4 Gy, only in A549 cells did the combination of Akt1 knockdown and rapamycin treatment enhance the number of residual γ-H2AX foci; there were approximately 40% more foci after the combined treatment compared to 28% after Akt1 knockdown alone.

Similar to the results achieved with the combined treatment of rapamycin/MK2206, combining rapamycin treatment with AKT1-siRNA led to a reduced number of foci in irradiated H460 cells (Fig 4C and 4D). Furthermore, effect of Akt inhibitor MK2206, rapamycin and the combination of MK2206 with rapamycin on NHEJ activity was tested in A549 cells stably expressing NHEJ platform. As shown in Fig 4E, MK2206 significantly inhibited repair of DSBs induced by I-SceI endonuclease. Treatment with rapamycin did not affect NHEJ repair while combination of rapamycin with MK2206 inhibited NHEJ that was markedly stronger than the effect of MK2206 alone.

In the *non-responder* MDA-MB-231 breast cancer cell line, knockdown of Akt1 by shRNA led to a significant increase in residual DNA-DSBs after irradiation with 2 Gy and with 4 Gy. Residual DSBs were also significantly increased when cells that had undergone shRNA mediated Akt1 knockdown were pre-treated with rapamycin. Treatment with rapamycin alone was not found to affect residual DNA-DSBs in cells that were examined at 24 h after irradiation. (Fig B in S4 Fig).

**Fig 5:**
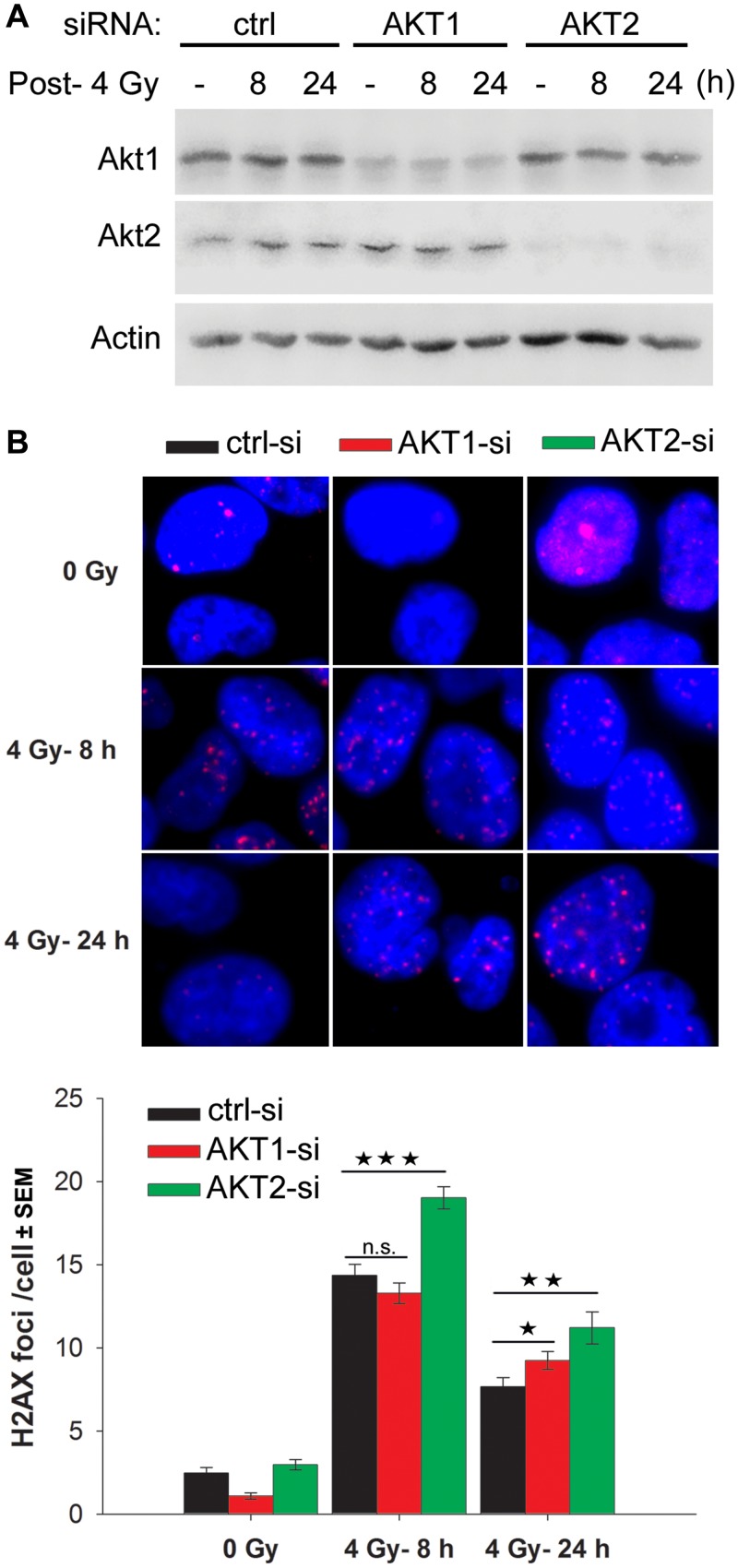
The effect of Akt2 on DNA-DSB repair is more robust than the effect of Akt1 in H460 cells. H460 cells that were grown on glass slides were transfected with 50 nM of control-siRNA (siCON), AKT1-siRNA (siAKT1) or AKT2-siRNA (siAKT2). Forty-eight hours (24 h time-point) and 64 h (8 h time-point) after transfection, cells were irradiated with 4 Gy and γ-H2AX assay was performed as described in *Materials and Methods*. The data represent the mean number of γ-H2AX foci ± SEM from at least 140 cells in two biologically independent experiments. Asterisks indicate statistically significant enhancement of residual γ-H2AX foci following transfection with AKT1- and AKT2-siRNA at the indicated conditions (*, P < 0.05; **, P < 0.01; ***, P < 0.001).

The data shown in Fig 3B indicate that Akt1 knockdown did not sensitize H460 cells to IR. However, the Akt inhibitor MK2206, which targets all 3 Akt isoforms, did induce significant radiosensitization (Fig 3A, S2 Fig). Based on these data, we hypothesized that in Akt1 knockdown H460 cells, DNA-DSB repair is regulated by the Akt2 and/or Akt3 isoforms. The data presented in Fig 5 support this hypothesis. As shown, the knockdown of Akt2 led to significantly increased residual DNA-DSBs at 8 h and 24 h after irradiation, whereas irradiated Akt1 knockdown cells exhibited only a slight increase in residual DNA-DSBs at 24 h post-irradiation (Fig 5).

Because rapamycin-mediated activation of Akt was shown to be dependent on PI3K activity (Fig 2C), we next investigated the effect of PI3K inhibition in combination with rapamycin on DNA-DSB repair in *non-responder* as well as in *responder* cells. To accomplish this, *non-responder* A549 cells were treated with rapamycin (500 nM), the PI3K inhibitor LY294002 (20 μM) or the combination of rapamycin and LY294002. At 24 h after a single dose of irradiation of 0 to 4 Gy, an analysis of residual DNA-DSBs using γ-H2AX foci assay revealed that pretreatment with LY294002 (but not with rapamycin) inhibited the repair of radiation-induced DNA-DSBs. Interestingly, after a combined pretreatment with LY294002 and rapamycin, the frequency of residual DNA-DSBs was significantly higher than after LY294002 treatment alone (Fig A in S5 Fig). To compare the effects produced by PI3K-dependent Akt activity on DNA-DSBs repair after rapamycin treatment in *responder* and *non-responder* cells, the effects produced by a combined treatment of rapamycin (100 and 500 nM) and LY294002 (10 μM) on DNA-DSBs repair were investigated. In agreement with the data shown in Fig A in S5 Fig, and in comparison to the effects produced by LY294002 alone, the combination treatment of LY294002 and rapamycin led to an increased number of residual γ-H2AX foci in *non-responder* A549 and SK-MES-1 cells that were irradiated with 4 Gy (Fig B in S5 Fig). This effect was more pronounced after increasing the rapamycin concentration from 100 nM to 500 nM. In contrast to the effects observed in *non-responder* cells, in *responder* H460 and H661 cells that were irradiated with 4 Gy, a combination of either 100 nM or 500 nM rapamycin with LY294002 (10 μM) led to a reduced number of γ-H2AX foci when compared to the effect produced by LY294002 alone (Fig B in S5 Fig).

## Discussion

Using a panel of five non-small cell lung cancer (NSCLC) cell lines and the breast cancer cell line MDA-MB-231, we demonstrated that the inhibition of mTORC1 by rapamycin leads to cell line-dependent radiosensitization. A lack of rapamycin-mediated radiosensitization was associated with the activation of the PI3K/Akt survival pathway. In cells that underwent radiosensitization in response to rapamycin (*responder* cells), the effect did not arise because of inhibition of the repair of radiation-induced DNA double-strand breaks (DNA-DSBs). However, in cells that were not radiosensitized in response to rapamycin (*non-responder* cells), increasing the activity of the PI3K/Akt pathway via pharmacological treatment led to an enhanced repair of DNA-DSBs and radioresistance. As a consequence, the combined targeting of Akt and mTORC1 restored the radiosensitizing effect of rapamycin in *non-responder* cells.

Previous studies have shown that activation of the PI3K/Akt survival pathway is one mechanism that leads to rapamycin resistance [33, 34]. In the present study, we demonstrated that using rapamycin to inhibit mTORC1 led to the activation of Akt in 3 out of 5 NSCLC cell lines and in the MDA-MB-231 cell line. Thus, rapamycin-mediated Akt activation is not restricted to tumor cells of specific origins. Existing reports have indicated that combining rapamycin (or its analogs) with radiotherapy results in tumor cell line-dependent radiosensitization. In agreement with previous reports [35–37], in the current study rapamycin-mediated the radiosensitization of only two of the five NSCLC cell lines that were tested. The lack of radiosensitization in the remaining NSCLC cell lines and in the MDA-MB-231 breast cancer cell line was associated with Akt activation following rapamycin treatment.

Akt activity is one of the major predictive markers for the radiation response. Akt phosphorylates, and, thus, inactivates pro-apoptotic proteins such as BAD and caspase-9 and it upregulates the anti-apoptotic proteins Bcl-2 and Bcl-XL. In addition to its anti-apoptotic function, Akt1 accelerates the repair of IR-induced DNA-DSBs through the interaction with DNA-PKcs [19, 21, 23]. Because in solid tumors apoptosis is only marginally involved in radiation toxicity [21, 38], we hypothesized that rapamycin-induced Akt activity leads to enhanced repair of IR-induced DNA-DSBs and consequently fosters radioprotection. Indeed, the data obtained from the γ-H2AX foci assays and NHEJ repair assay support this conclusion. Combining PI3K/Akt targeting with rapamycin treatment led to an increased frequency of residual DNA-DSBs only in *non-responder* cells (i.e., not radiosensitized by rapamycin) when compared with PI3K/Akt targeting strategies alone. This observation indicates that rapamycin-mediated Akt activation stimulates the repair of DNA-DSBs that are induced by ionizing radiation. Because a linear correlation exists between the frequency of residual DNA-DSBs and radiosensitivity [39], we propose that rapamycin-mediated Akt activation leads to enhanced post-irradiation cell survival. There is no direct correlation between rapamycin-mediated Akt phosphorylation and phosphorylation of Akt substrates, especially GSK3α (see Fig 2A). This indicates that the lack of radiosensitization by rapamycin in *non-responder* cells is independent of the function of Akt substrate GSK3, but depends on the alternative role of Akt, which is stimulating repair of DSB through NHEJ [40]. In *responder* cells, the combination of rapamycin and Akt targeting reduced the number of residual DNA-DSBs. At present, the underlying mechanism for this observation is not clear. Akt1 knockdown in the absence of rapamycin in both *non-responder* A549 cells and *responder* H460 cells led to an increased frequency of residual DNA-DSBs. Compared to H460 cells, this effect was stronger in A549 cells. However, the increased number of residual DNA-DSBs after Akt1 knockdown was associated with radiosensitization in A549 cells, but not in H460 cells. Because inhibiting Akt via treatment with MK2206 led to radiosensitization in H460 cells (see Fig 3A & S2 Fig), the lack of radiosensitization produced by AKT1-siRNA in H460 cells (see Fig 3B) may indicate that other Akt isoforms (e.g., Akt2) are dominant in this cell line. Thus, Akt2 may compensate for the functions of Akt1 following its siRNA-mediated knockdown. This interpretation is supported by the much stronger effect that was produced on the inhibition of DNA-DSB repair following Akt2 knockdown versus Akt1 knockdown after irradiation (Fig 5). According to our results, rapamycin appears to be a potential radiosensitizer of solid tumor cells, such as NSCLC cells and breast cancer cells. However, in *non-responder* tumor cells, the radiosensitizing effect of rapamycin cannot be shown, and the activation of the PI3K/Akt survival pathway occurs in a rapamycin-mediated manner. It has been shown that the activation of PI3K/Akt accelerates the repair of radiation-induced DNA-DSBs [19, 22, 41] through NHEJ [11, 23]. Thus, rapamycin mediated activation of the PI3K/Akt pathway can lead to post-irradiation cell survival as a result of the stimulation of DNA-DSBs repair, which interferes with the radiosensitizing effect of rapamycin. According to this model, the radiosensitizing effect of rapamycin in *non-responder* cells can be masked by Akt-mediated radioresistance. Under this condition, improved radiosensitizing effect of rapamycin by Akt targeting can be discussed as a synthetic lethality approach. In *responder* cells, due to a lack of rapamycin-mediated stimulation of Akt, radiosensitization by rapamycin can occur.

Thus far, how mTORC1 inhibitors work to enhance radiation toxicity in cancer cells has not been well defined. Chen et al. [42] demonstrated for the first time that rapamycin suppresses DNA double-strand break repair in breast cancer MCF7 cells *in vitro*. In the present study, a universal phenotype of impaired repair of radiation-induced DNA-DSBs by rapamycin was not indicated in any of the examined cell lines. Therefore, the effect reported by Chen et al. [42] might have been a cell line-dependent phenotype of MCF7 cells.

In recent years, autophagy has been recognized as an important process in mediating the response of tumor cells to therapy, especially, radiation therapy. Exposure to ionizing radiation induces autophagy and protects tumor cells against irradiation. Thus, as it has been described by others and by us, the inhibition of autophagy either by pharmacological inhibitors or genetic approaches induces radiosensitization of human solid tumor cells [43–45]. In contrast to the cytoprotective effects produced by radiation-induced autophagy, an induction of autophagy via the PI3K/Akt/mTOR pathway has been reported to have a cytotoxic effect [46]. Because rapamycin is an effective inducer of autophagy, the radiosensitizing effect of rapamycin might be due to the induction of autophagy rather than impaired repair of DNA-DSBs. In the current study, we demonstrated that, out of 6 cell lines that were tested, rapamycin treatment does not impair the repair of radiation-induced DNA-DSBs, except for SK-MES-1 when treated at a very high concentration of 500 nM. Furthermore, in support of the association between rapamycin-induced autophagy and radiosensitization, Nam *et al*. [47] reported that prolonged autophagy in response to mTORC1 inhibition drives radioresistant cancer cells into senescence [47].

The potential mechanism(s) of rapamycin-mediated Akt activation has/have not been described. P53 interacts with Akt and is important for cell survival [48]. Mutational status of PI3K, p53, K-RAS and EGFR in 5 NSCLC cell lines used in the current study have been reported earlier [49]. In the present study, rapamycin could induce Akt activation differentially in A549 and H460 cells, both expressing wild type p53 [50]. In both of these cell lines, the level of radiation-induced expression of p53 was quite similar [50]. These data, in line with a report by Nagata *et al*. [51] that demonstrated that mTORC1 targeting by rapamycin is a promising strategy for radiosensitization and has no reliance on p53 gene status [51], suggest that rapamycin-mediated activation of Akt is p53-independent.

Akt and DNA-PKcs physically interact with each other and lead to the activation of DNA-PKcs by Akt, and vice versa [21, 23, 52]. Li *et al*. reported that protein phosphatase 2A (PP2A) and DNA-PKcs are both involved in mediating rapamycin-induced Akt phosphorylation [53]. We have previously shown similar levels of expression and activation of DNA-PKcs in rapamycin *responder* (e.g., H460) and rapamycin *non-responder* (e.g., A549) cells without rapamycin treatment [21]. Thus, differential activation of Akt following rapamycin treatment does not appear to result from the differential expression of DNA-PKcs, per se. Further studies are necessary to evaluate the genetic status and expression patterns of the components of the previously described pathways that are involved in Akt activation after rapamycin treatment. Among these components, IRS1, P70S6K and PP2A are thus far the most important candidates for the identification of predictive biomarker(s) for rapamycin-mediated Akt activation.

## Conclusion

**Fig 6:**
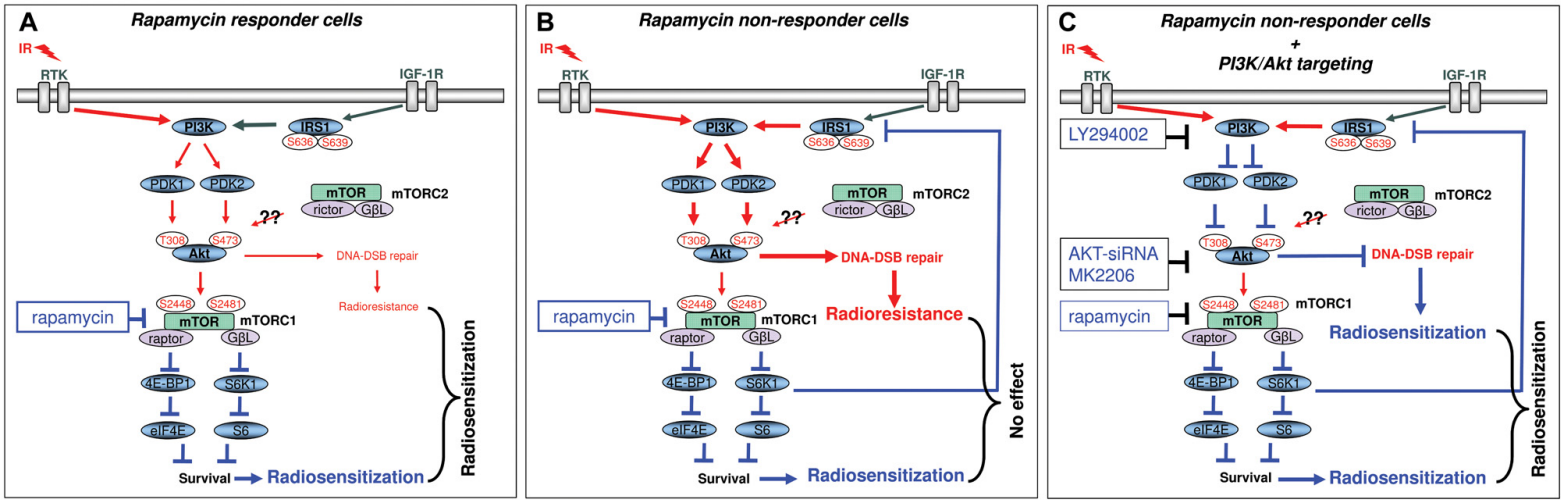
Schematics illustrating the potential pathways by which dual targeting of Akt and mTORC1 enhances the frequency of residual DNA-DSBs and increases radiation sensitivity.

As summarized in Fig 6, the inhibition of mTORC1 with rapamycin mediates cell line-dependent activation of the Akt pathway. Akt activity accelerates repair of ionizing radiation-induced DNA-DSB, which leads to resistance to radiosensitization by rapamycin. Thus, targeting of Akt will be a means for improving radiosensitization by mTORC1 inhibitor rapamycin through enhanced frequency of residual DNA-DSBs. This approach may offer clinical potential towards improving radiotherapy outcomes of radioresistant solid tumors. Further animal studies are warranted to determine the association between Akt activation and rapamycin treatment and the effects produced by the combination of rapamycin and irradiation on local tumor control.

## Supporting Information

S1 Fig Antiproliferative effect of rapamycin in NSCLC cells. Cells (3 x 10^4^) were plated in 6 cm culture dishes and were treated with or without rapamycin at 100 nM, 200 nM or 500 nM after 24 hours. At the indicated days after treatment, cells were counted and graphed. The data points represent the mean cell counts ± SD of 4 parallel experiments.
(PPTX)

S2 Fig Radiosensitization of NSCLC cells by different concentrations of MK2206. Cells were plated in 6-well plates and were treated after 24 h with the indicated concentrations MK2206 for 3 hours. Thereafter, cells were either mock irradiated or irradiated with 3 Gy and incubated to facilitate colony formation. Clonogenic assays were performed as described in *Materials and Methods*. The data represent the mean SF ± SEM of two biologically independent experiments; each experiment contained six parallel data sets.
(PPTX)

S3 Fig Akt targeting promotes the radiosensitizing effect of rapamycin in *non-responder* cells. Cells were plated in 6-well plates and were treated after 24 h with with MK2206 (5 μM) for 1 h, followed by treatment with rapamycin (100 nM) for 2 h. Control cells received the appropriate concentrations of DMSO. The cultures were irradiated after rapamycin treatment and incubated for colony growth. Data represent the mean SF ± SD of 6 parallel experiments.
(PPTX)

S4 Fig Akt1 knockdown in combination with rapamycin promotes the radiosensitizing effect of rapamycin and leads to an increased frequency of non-repaired DNA-DSBs in MDA-MB-231 cells. Akt1 knockdown was analyzed in MDA-MB-231 cells that were stably transfected with either scramble shRNA (shSCR) or AKT1-shRNA (shAKT1) by Western blotting. GAPDH was used as a loading control. Densitometry data represent the mean ratio of Akt1 to GAPDH based on two biologically independent experiments. For the colony formation assay, cells were plated in culture dishes and were treated after 24 hours with rapamycin (100 nM) for 2 hours. Thereafter, cells were either mock irradiated or irradiated with the indicated doses of IR and incubated to facilitate colony formation. Clonogenic assays were performed as described in *Materials and Methods*. The data represent the mean SF ± SD of three biologically independent experiments; each experiment contained six parallel data sets. Asterisks indicate a significant difference between the radiosensitizing effect produced by rapamycin in AKT1-shRNA cells compared to the effect produced by AKT1-shRNA alone (*, P < 0.05, Student's *t-*test) **(Fig A)**. MDA-MB-231 cells, stably transfected with scramble-shRNA (shSCR) or AKT1-shRNA (shAKT1), were grown on glass slides and then treated with rapamycin (100 nM) for 3 hours. Thereafter, cells were either mock irradiated or irradiated with the indicated doses of X-ray. γ-H2AX foci assays were performed and the frequency of residual γ-H2AX foci was counted 24 hours after irradiation, as described in *Materials and Methods*. The data represent the mean number of γ-H2AX foci ± SEM in 100 to 150 counted cells from three biologically independent experiments. Asterisks indicate a statistically significant difference in the number of residual γ-H2AX foci between the indicated conditions (*, P < 0.05; ***, P < 0.001, Student's *t-*test) **(Fig B)**.
(PPTX)

S5 Fig Combination of PI3K inhibitor LY294002 with rapamycin enhances the residual DNA-DSBs only in *non-responder* cells. A549 cells were grown to confluency on glass slides and concurrently treated with LY294002 (20 μM) and rapamycin (500 nM) or pretreated with LY294002 (20 μM) for 1 hour and followed by treatment with rapamycin (500 nM) for 2 h **(Fig A)**. The indicated confluent cells, which were grown on glass slides, were treated with LY294002 (10 μM) and the indicated concentrations of rapamycin (100 or 500 nM) or pretreated with LY294002 (10 μM) for 1 hour and followed by treatment with rapamycin (100 or 500 nM) for 2 h. Thereafter, cells were either mock irradiated or irradiated with the indicated doses of X-ray. γ-H2AX foci assays were performed and the frequency of residual γ-H2AX foci was counted 24 hours after irradiation, as described in *Materials and Methods*. Asterisks indicate a statistically significant difference in the number of residual γ-H2AX foci between the indicated conditions (*, P < 0.05; **, P < 0.01; ***, P < 0.001, Student's *t-*test) **(Fig B)**.
(PPTX)

S1 Table Rapamycin prolongs the population doubling time of NSCLC cells. Cells were plated in 6 cm culture tissues and were treated after 24 h with the indicated concentrations of rapamycin. Control cells received the appropriate concentrations of DMSO. To calculate the population doubling time (PDT) of each group, mean cell counts were taken at day 4 after treatment with rapamycin or control. The effect of rapamycin on PDT was calculated by determining the ratios of PDTs in rapamycin (rapa) treated cells versus the control (ctrl).
(PPTX)
