# Supplementary figures and images for: Dual Targeting of Akt and mTORC1 Impairs Repair of DNA Double-Strand Breaks and Increases Radiation Sensitivity of Human Tumor Cells
Source: PLoS One. 2016 May 3;11(5):e0154745. doi: 10.1371/journal.pone.0154745 (PMC4854483; doi:10.1371/journal.pone.0154745)

## Slide 1
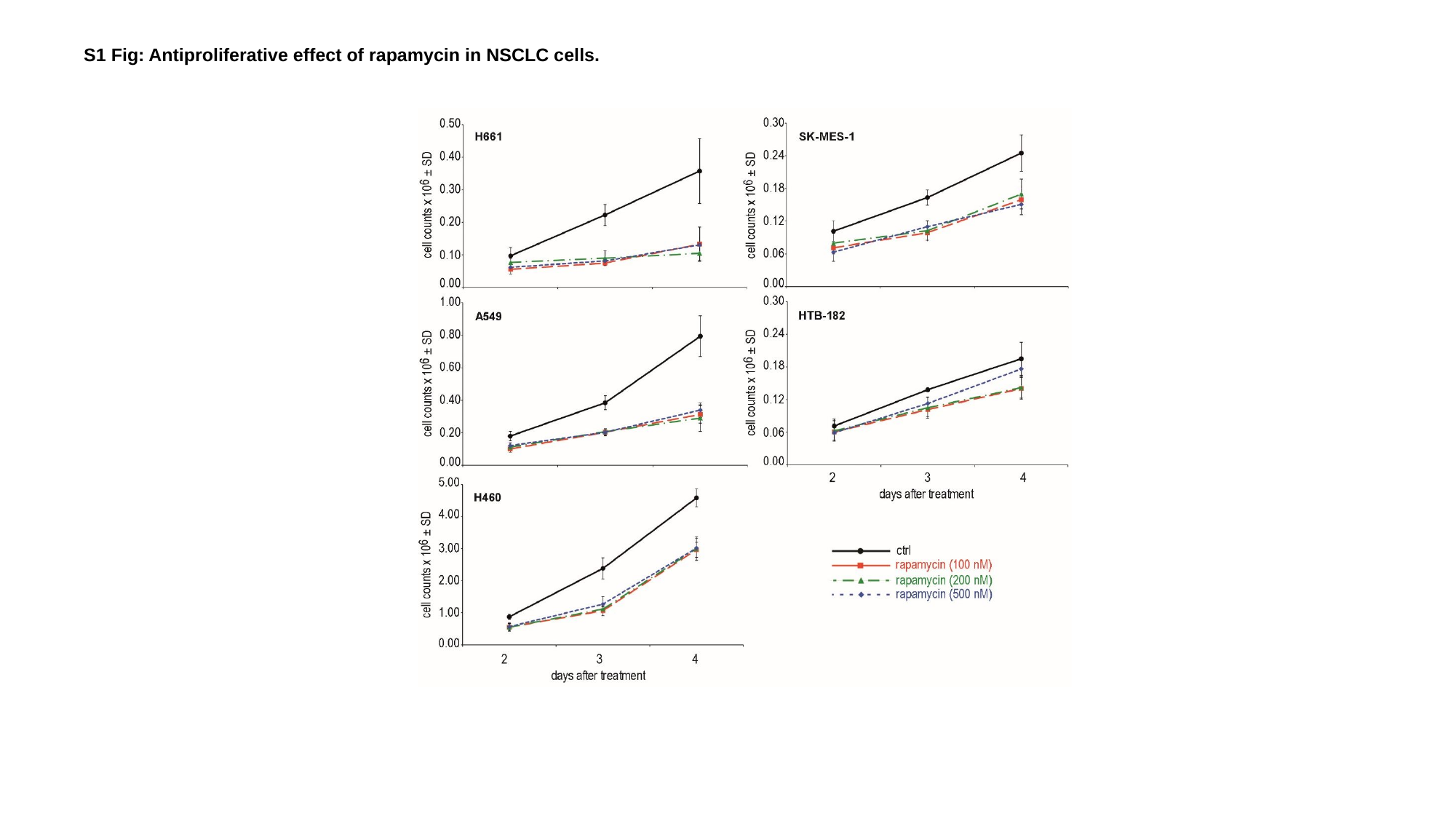

S1 Fig: Antiproliferative effect of rapamycin in NSCLC cells.

Supplement: S1 Fig — Cells (3 x 10^4^) were plated in 6 cm culture dishes and were treated with or without rapamycin at 100 nM, 200 nM or 500 nM after 24 hours. At the indicated days after treatment, cells were counted and graphed. The data points represent the mean cell counts ± SD of 4 parallel experiments. (PPTX) [file pone.0154745.s001.pptx]

## Slide 1
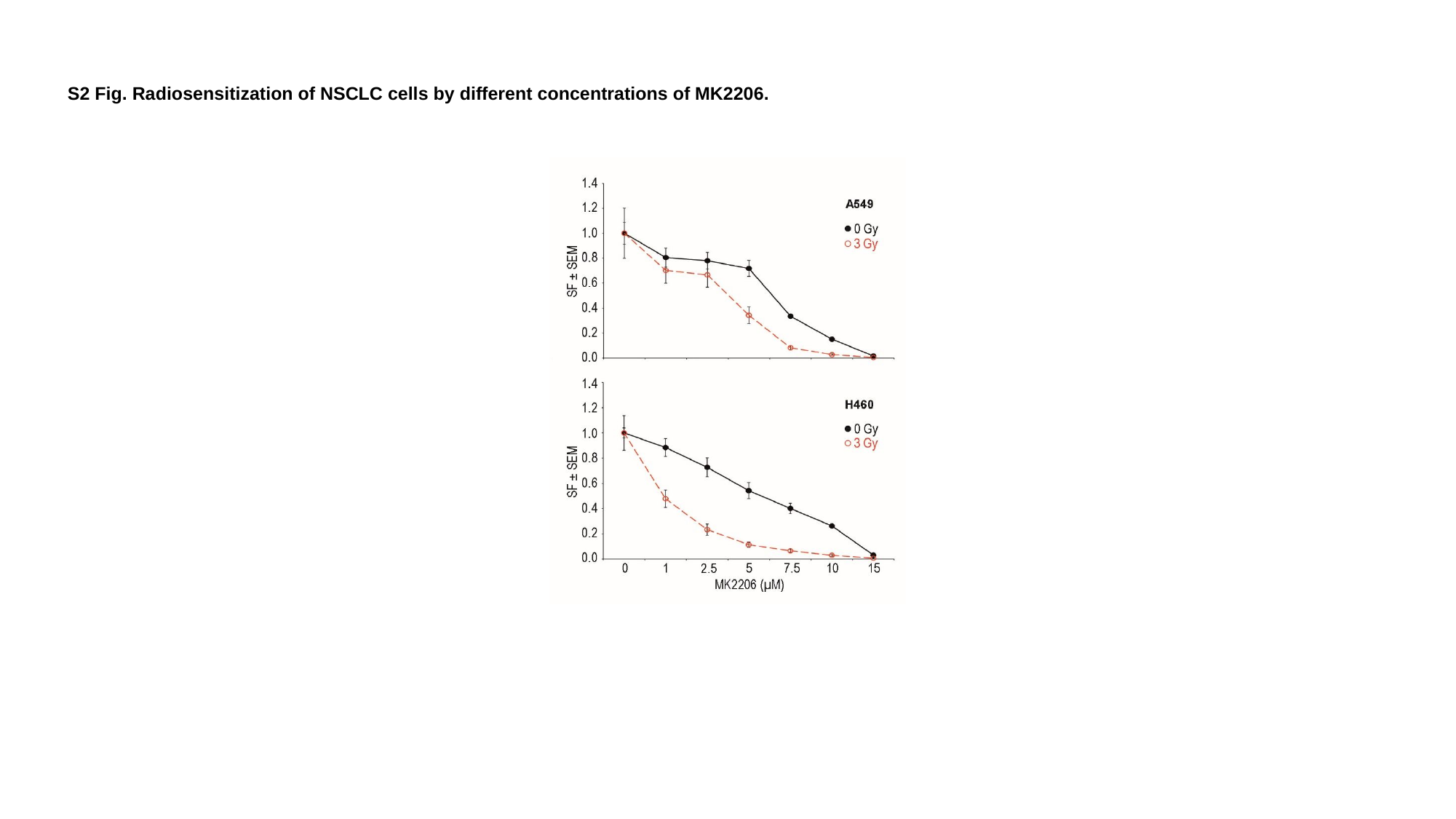

S2 Fig. Radiosensitization of NSCLC cells by different concentrations of MK2206.

Supplement: S2 Fig — Cells were plated in 6-well plates and were treated after 24 h with the indicated concentrations MK2206 for 3 hours. Thereafter, cells were either mock irradiated or irradiated with 3 Gy and incubated to facilitate colony formation. Clonogenic assays were performed as described in Materials and Methods. The data represent the mean SF ± SEM of two biologically independent experiments; each experiment contained six parallel data sets. (PPTX) [file pone.0154745.s002.pptx]

## Slide 1
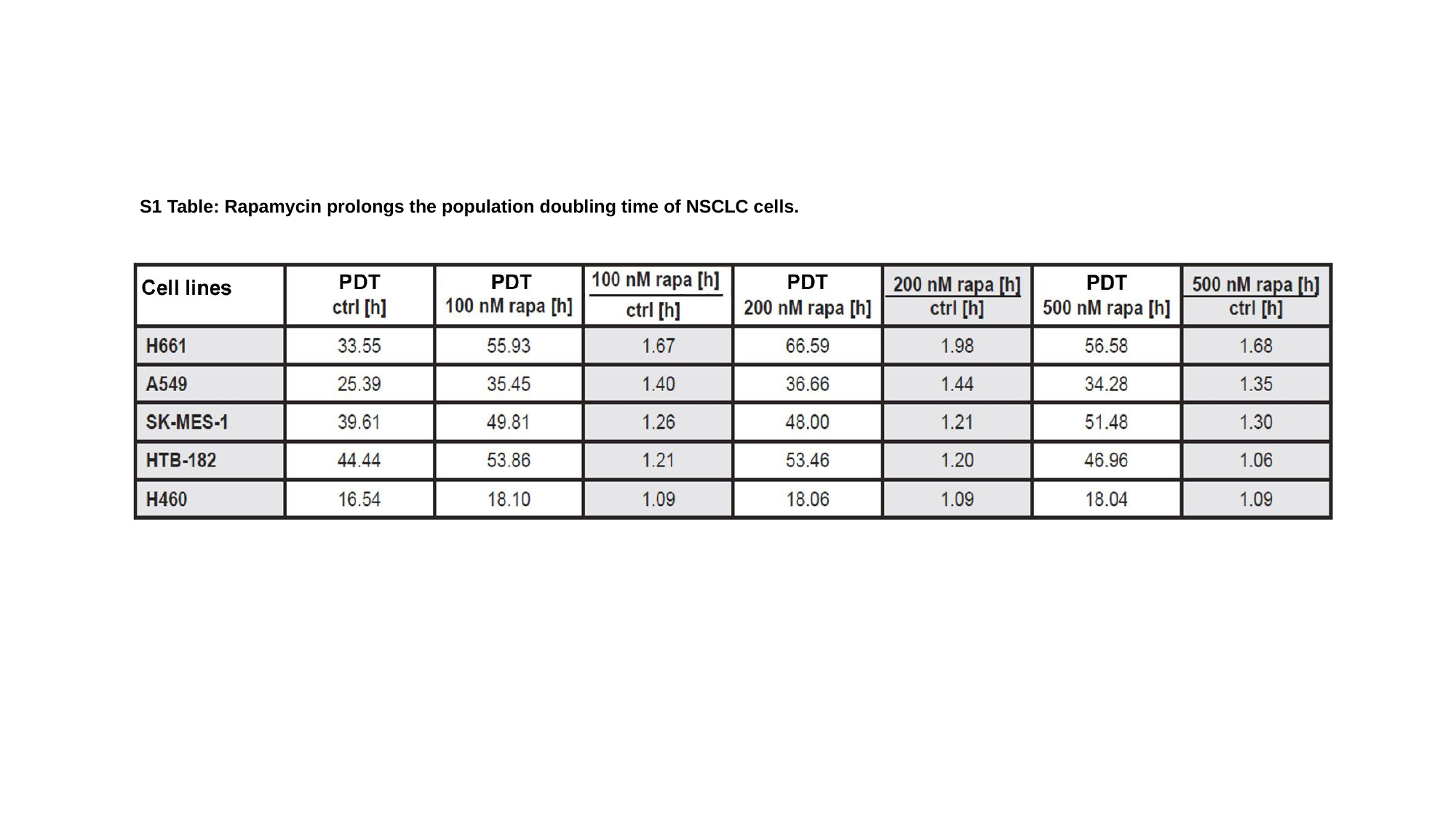

S1 Table: Rapamycin prolongs the population doubling time of NSCLC cells.

Supplement: S1 Table — Cells were plated in 6 cm culture tissues and were treated after 24 h with the indicated concentrations of rapamycin. Control cells received the appropriate concentrations of DMSO. To calculate the population doubling time (PDT) of each group, mean cell counts were taken at day 4 after treatment with rapamycin or control. The effect of rapamycin on PDT was calculated by determining the ratios of PDTs in rapamycin (rapa) treated cells versus the control (ctrl). (PPTX) [file pone.0154745.s006.pptx]
