# Supplementary material for: Dual Targeting of Akt and mTORC1 Impairs Repair of DNA Double-Strand Breaks and Increases Radiation Sensitivity of Human Tumor Cells
Source: PLoS One. 2016 May 3;11(5):e0154745. doi: 10.1371/journal.pone.0154745 (PMC4854483; doi:10.1371/journal.pone.0154745)
Supplement: S3 Fig — Cells were plated in 6-well plates and were treated after 24 h with with MK2206 (5 μM) for 1 h, followed by treatment with rapamycin (100 nM) for 2 h. Control cells received the appropriate concentrations of DMSO. The cultures were irradiated after rapamycin treatment and incubated for colony growth. Data represent the mean SF ± SD of 6 parallel experiments. (PPTX) [file pone.0154745.s003.pptx]

## Slide 1
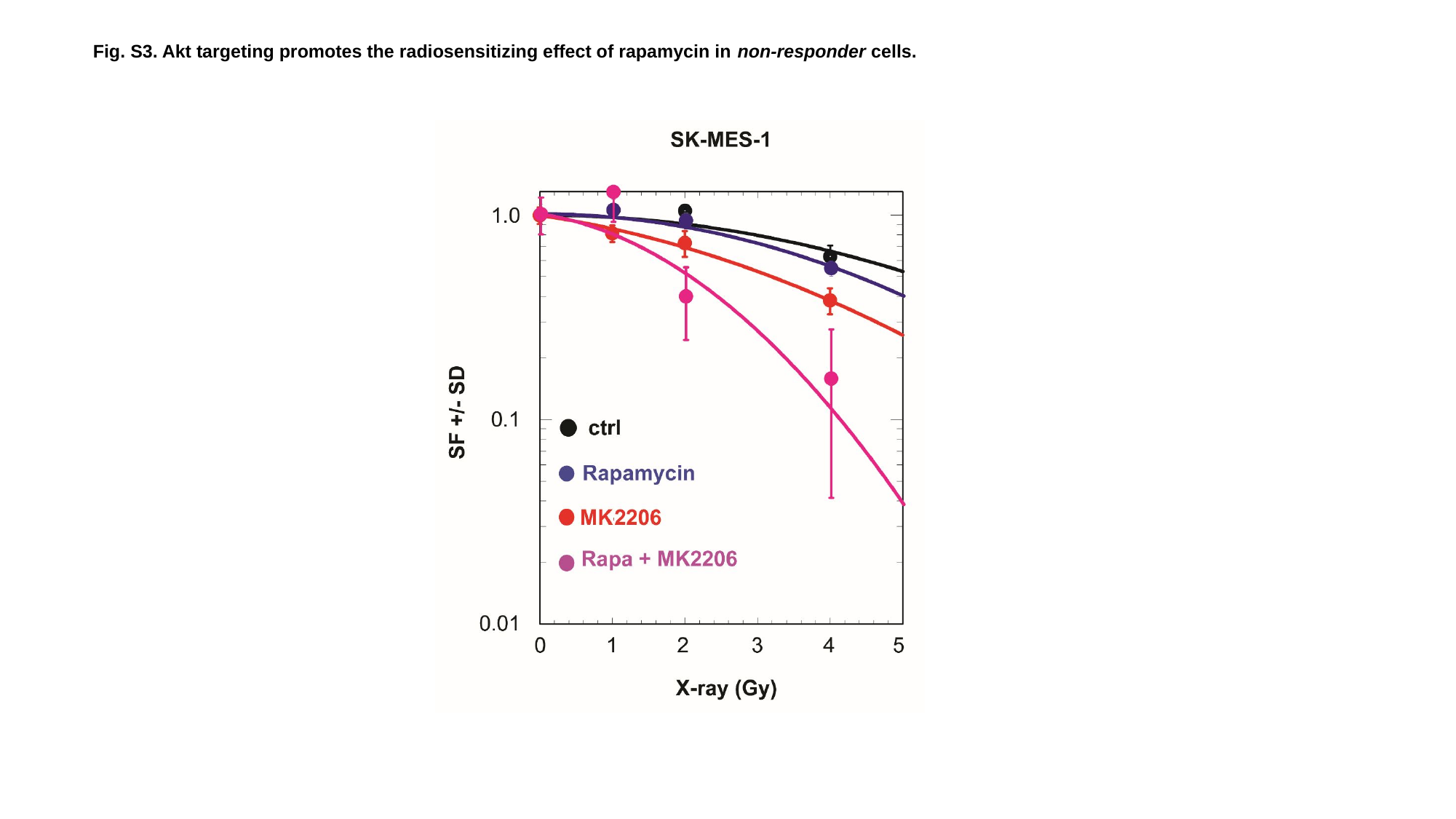

Fig. S3. Akt targeting promotes the radiosensitizing effect of rapamycin in non-responder cells.
