# Supplementary material for: Dual Targeting of Akt and mTORC1 Impairs Repair of DNA Double-Strand Breaks and Increases Radiation Sensitivity of Human Tumor Cells
Source: PLoS One. 2016 May 3;11(5):e0154745. doi: 10.1371/journal.pone.0154745 (PMC4854483; doi:10.1371/journal.pone.0154745)
Supplement: S4 Fig — Akt1 knockdown was analyzed in MDA-MB-231 cells that were stably transfected with either scramble shRNA (shSCR) or AKT1-shRNA (shAKT1) by Western blotting. GAPDH was used as a loading control. Densitometry data represent the mean ratio of Akt1 to GAPDH based on two biologically independent experiments. For the colony formation assay, cells were plated in culture dishes and were treated after 24 hours with rapamycin (100 nM) for 2 hours. Thereafter, cells were either mock irradiated or irradiated with the indicated doses of IR and incubated to facilitate colony formation. Clonogenic assays were performed as described in Materials and Methods. The data represent the mean SF ± SD of three biologically independent experiments; each experiment contained six parallel data sets. Asterisks indicate a significant difference between the radiosensitizing effect produced by rapamycin in AKT1-shRNA cells compared to the effect produced by AKT1-shRNA alone (*, P < 0.05, Student's t-test) (Fig A). MDA-MB-231 cells, stably transfected with scramble-shRNA (shSCR) or AKT1-shRNA (shAKT1), were grown on glass slides and then treated with rapamycin (100 nM) for 3 hours. Thereafter, cells were either mock irradiated or irradiated with the indicated doses of X-ray. γ-H2AX foci assays were performed and the frequency of residual γ-H2AX foci was counted 24 hours after irradiation, as described in Materials and Methods. The data represent the mean number of γ-H2AX foci ± SEM in 100 to 150 counted cells from three biologically independent experiments. Asterisks indicate a statistically significant difference in the number of residual γ-H2AX foci between the indicated conditions (*, P < 0.05; ***, P < 0.001, Student's t-test) (Fig B). (PPTX) [file pone.0154745.s004.pptx]

## Slide 1
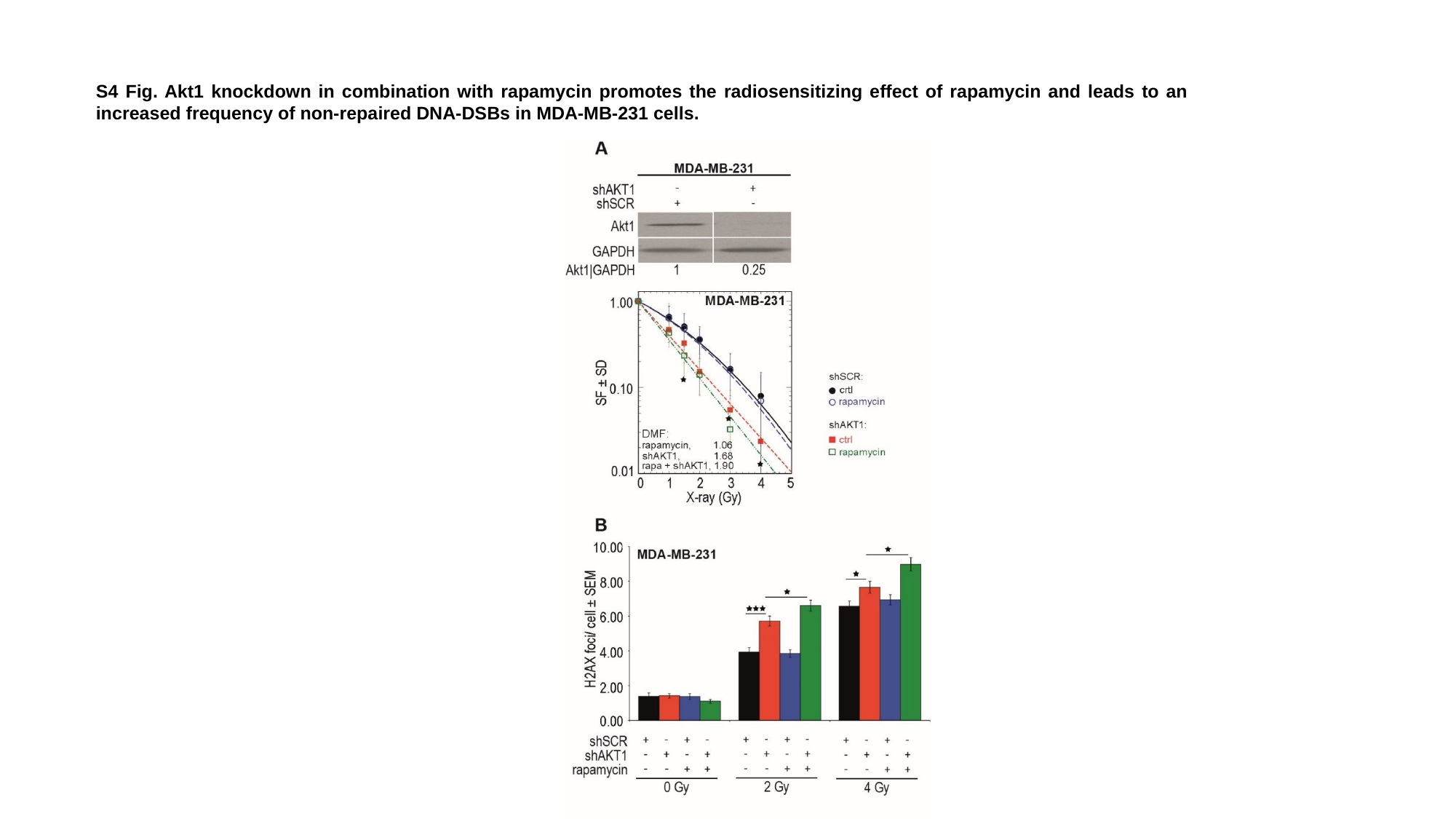

S4 Fig. Akt1 knockdown in combination with rapamycin promotes the radiosensitizing effect of rapamycin and leads to an increased frequency of non-repaired DNA-DSBs in MDA-MB-231 cells.
