# Supplementary material for: Dual Targeting of Akt and mTORC1 Impairs Repair of DNA Double-Strand Breaks and Increases Radiation Sensitivity of Human Tumor Cells
Source: PLoS One. 2016 May 3;11(5):e0154745. doi: 10.1371/journal.pone.0154745 (PMC4854483; doi:10.1371/journal.pone.0154745)
Supplement: S5 Fig — A549 cells were grown to confluency on glass slides and concurrently treated with LY294002 (20 μM) and rapamycin (500 nM) or pretreated with LY294002 (20 μM) for 1 hour and followed by treatment with rapamycin (500 nM) for 2 h (Fig A). The indicated confluent cells, which were grown on glass slides, were treated with LY294002 (10 μM) and the indicated concentrations of rapamycin (100 or 500 nM) or pretreated with LY294002 (10 μM) for 1 hour and followed by treatment with rapamycin (100 or 500 nM) for 2 h. Thereafter, cells were either mock irradiated or irradiated with the indicated doses of X-ray. γ-H2AX foci assays were performed and the frequency of residual γ-H2AX foci was counted 24 hours after irradiation, as described in Materials and Methods. Asterisks indicate a statistically significant difference in the number of residual γ-H2AX foci between the indicated conditions (*, P < 0.05; **, P < 0.01; ***, P < 0.001, Student's t-test) (Fig B). (PPTX) [file pone.0154745.s005.pptx]

## Slide 1
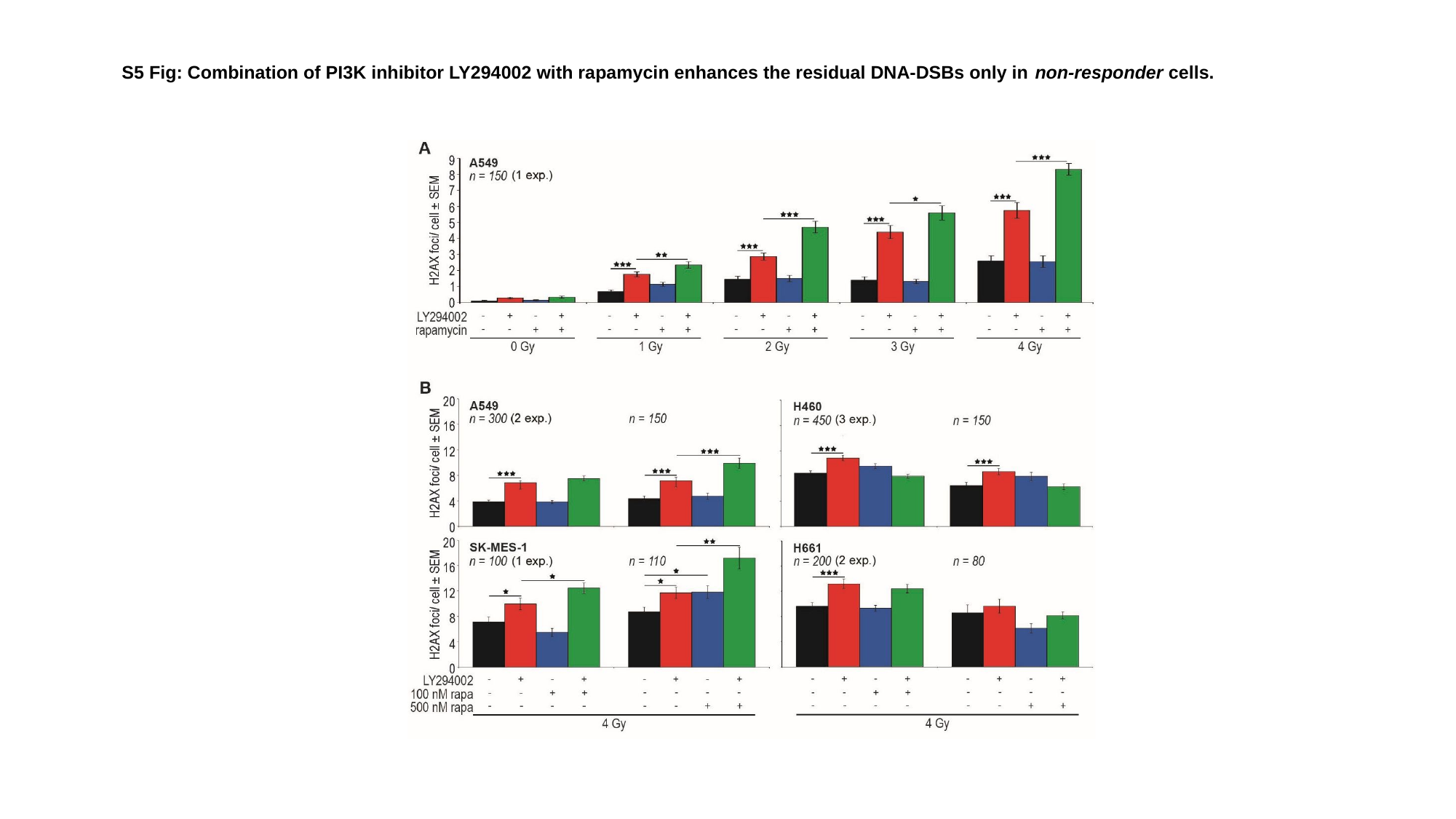

S5 Fig: Combination of PI3K inhibitor LY294002 with rapamycin enhances the residual DNA-DSBs only in non-responder cells.
